# Socioeconomic Differences in Dietary Patterns in an East African Country: Evidence from the Republic of Seychelles

**DOI:** 10.1371/journal.pone.0155617

**Published:** 2016-05-23

**Authors:** Ana-Lucia Mayén, Pascal Bovet, Helena Marti-Soler, Bharathi Viswanathan, Jude Gedeon, Fred Paccaud, Pedro Marques-Vidal, Silvia Stringhini

**Affiliations:** 1 Institute of Social and Preventive Medicine (IUMSP), Lausanne University Hospital, Lausanne, Switzerland; 2 Ministry of Health, Victoria, Republic of Seychelles; 3 Department of Medicine, Internal Medicine, Lausanne University Hospital, Lausanne, Switzerland; St Francis Hospital, UNITED STATES

## Abstract

**Background:**

In high income countries, low socioeconomic status (SES) is related to unhealthier dietary patterns, while evidence on the social patterning of diet in low and middle income countries is scarce.

**Objective:**

In this study, we assess dietary patterns in the general population of a middle income country in the African region, the Republic of Seychelles, and examine their distribution according to educational level and income.

**Methods:**

Data was drawn from two independent national surveys conducted in the Seychelles among adults aged 25–64 years in 2004 (n = 1236) and 2013 (n = 1240). Dietary patterns were assessed by principal component analysis (PCA). Educational level and income were used as SES indicators. Data from both surveys were combined as no interaction was found between SES and year.

**Results:**

Three dietary patterns were identified: “snacks and drinks”, “fruit and vegetables” and “fish and rice”. No significant associations were found between SES and the “snacks and drinks” pattern. Low vs. high SES individuals had lower adherence to the “fruit and vegetables” pattern [prevalence ratio (95% CI) 0.71 (0.60–0.83)] but a higher adherence to the traditional “fish and rice” pattern [1.58 (1.32–1.88)]. Income modified the association between education and the “fish and rice” pattern (p = 0.02), whereby low income individuals had a higher adherence to this pattern in both educational groups.

**Conclusion:**

Low SES individuals have a lower consumption of fruit and vegetables, but a higher consumption of traditional foods like fish and rice. The Seychelles may be at a degenerative diseases stage of the nutrition transition.

## Introduction

In high income countries (HICs), socioeconomic status (SES) is a major determinant of healthy eating: individuals with a high SES tend to have a higher intake of whole grains, lean meat and fish, low-fat dairy products, fresh vegetables and fruit [1], while individuals with a low SES have a higher intake of refined grains and fatty foods [2–4]. A western-like diet, low in fiber and rich in fat, salt and sugar, has been associated with an increased body mass index (BMI) and non-communicable diseases (NCD) development [5]. Thus, the higher prevalence of NCDs in low SES individuals may be partly explained by unhealthier dietary patterns in this group. The effect of SES on diet may be related to several factors. For example, low SES individuals are more exposed to adverse psychosocial conditions such as stress at home or work settings, and lack social networks and support [6, 7], which may lead to the adoption of unhealthy behaviours (e.g. smoking, high fat diets, heavy drinking) [8–11]. Low SES individuals may also have less knowledge about healthy behaviours, health risks [8, 12], and be less willing to invest in future health [13]. Additionally, low SES individuals may have a lower access to more expensive healthy foods because of financial and environmental constraints [14–16].

In low and middle income countries (LMICs), shifts in physical activity, and the structure and composition of diet are occurring along with demographic and socioeconomic changes. Fiber-rich foods are being substituted by foods high in fat and sugar while physical activity levels are decreasing. These changes are defined as the nutrition transition theory [17, 18]. Further, evidence on social differences in diet in LMICs is scarce. The few studies that have examined the social patterning of dietary intake have generally found that wealthier populations have an unhealthier diet, higher in cholesterol and saturated fats and lower in fiber [10]. Possible reasons include a higher access to processed foods available in supermarkets and the adoption of “western” dietary patterns by the upper classes [17, 19]. In spite of this, there is evidence to suggest that the social patterning of diet may reverse over time along with socioeconomic development, with poverty-stricken populations adopting unhealthier diets at later stages of the nutrition transition [20, 21]. This reversal has been shown before for obesity [21] and cardiovascular risk factors [22].

Almost three quarters of worldwide NCD-related deaths occur in LMICs [23]. As diet is one of the major modifiable risk factors for NCDs, several aspects of diet are included in the nine targets of the World Health Organization Global Action Plan for the Prevention and Control of NCDs 2013–2020 to reduce NCD mortality by 25% by 2025 [24]. Evidence on the social distribution of dietary patterns is essential for an adequate implementation of this action plan.

Only few studies have assessed the social distribution of diet in African countries [25–30], and some were conducted among high SES individuals only [31, 32]. These studies have generally shown that individuals with high SES have a higher intake of fruit and vegetables [30] and a lower intake of traditional foods compared to individuals with low SES [25, 26]. Few studies have assessed diet in Seychelles and they show an increasing intake of calories, salt and vegetable oils [33], plus a decreasing intake of traditional foods [34]. However, no study has assessed the social distribution of dietary patterns in Seychelles. In this study, we assessed dietary patterns in the general population of the Republic of Seychelles, a higher middle income country in the African region, and examine their distribution according to educational level and income. We hypothesize that, in the Seychelles, individuals with lower SES have an unhealthier western-like diet compared to those with higher SES, as observed in HICs.

## Materials and Methods

### Study population

The Republic of Seychelles is a country located in the Indian Ocean with a total population of 84,000 in 2004 and 93,000 in 2013 [35]. The large majority of inhabitants are of African descent with minorities from Caucasian, Indian or Chinese descent [36]. While based only 50 years ago on the exportation of coprah, cinnamon and other cash crops, Seychelles rising economy currently relies on tourism, industrial fishing, and services [37]. The gross national income (GNI) per capita increased from 2,000 US$ in the 1980s to more than 13,000 US$ in 2014 [38]. Since more than a decade ago, nearly all children have attended at least 10 years of free and compulsory education (kindergarten, primary and secondary) up to the age of 15–16 years [39]. At least one third of the population attends post secondary vocational or academic education. The unemployment rate for men and women has been low for several years, e.g. <5% in 2014 [40]. A high prevalence of cardiovascular risk factors (CVRFs) has been previously described, including a high and increasing prevalence of overweight and obesity [33].

Two cross sectional national surveys of CVRFs were conducted in 2004 (n = 1236) and 2013 (n = 1240), including participants aged 25–64 years. The sampling methods have been described previously [41, 42]. Briefly, for each survey, eligible individuals were selected from a database derived from population censuses, regularly updated by civil authorities. Random samples were drawn from the entire population. Eligible participants were invited to attend the survey centers and the ones who did not attend were traced and re-invited to attend. The participation rate in 2004 was 80.3% and in 2013 was 72.9%. As associations between SES and dietary patterns were similar in the two surveys (p for interaction between SES and year >0.05) and the sample size was rather small, we pooled data from the two surveys. The surveys were approved by the research and ethical board of the Ministry of Health of the Republic of Seychelles and all participants provided an informed written consent.

### Measures

#### Socioeconomic status (SES)

We used two indicators of SES in this study: education and income. Education refers to the highest level of education attained. The variable was categorized in two groups: “high” (post secondary school: polytechnic school, university or similar schools, excluding post secondary vocational school) and “low” (secondary school and post secondary vocational school). Income refers to average personal earnings per month in the Seychelles Rupees. Income substantially differed between 2004 and 2013, partly because of an economic reform. Thus we harmonized the variable by creating two categories: low income (≤3,000 rupees in 2004 and ≤8,000 in 2013) and high income (>3,000 rupees in 2004 and >8,000 rupees in 2013) to have similar proportions of the population in each group for both years.

#### Dietary intake

Food frequency questionnaires (FFQs) were used to assess dietary intake. These were prepared in collaboration with the Nutrition Unit of the Ministry of Health in Seychelles, taking into account local dietary habits. The questionnaires assessed the number of days per week certain food items were consumed (e.g. fruit, rice, salad, fish, chicken, etc) [43, 44]. Participants were asked about their retrospective weekly intake of selected food items and those with missing values for at least one food group (e.g. vegetables) were excluded from the analytical sample (n = 9 in 2004 and n = 0 in 2013). The FFQs included between 25 and 33 foods for both surveys and were grouped into 18 foods, which appear in S1 Table. As the FFQs for both years were slightly different, categories for fruit, fish, meat, drinks and juice were harmonized before merging dietary intake data.

### Statistical analysis

Analyses were conducted using Stata version 13.1 (Stata Corp., College Station, TX, USA). Dietary patterns were obtained using principal component analysis (PCA) with varimax orthogonal rotation [45] to minimize correlation between components [46]. This is a widely used statistical method to identify dietary patterns [47] and we expect a variance similar to other studies to explain variability of the data [48–50]. A Kaiser-Meyer-Olking (KMO) test was performed to assess the adequacy of the data for PCA and all food items with a value >0.5 were included. Two criteria were used to retain dietary patterns after running the PCA [51, 52]: an eigenvalue higher than one and the interpretability of each dietary pattern. Food items with an absolute factor loading greater than 0.2 were considered to characterize the dietary pattern. Dietary patterns were named after the food items with the highest factor loadings (i.e. those with the highest correlation to the dietary patterns). A dietary score was assigned to each participant for each of the three dietary patterns, to indicate the accordance between the participant’s usual intake and each food pattern. Scores were calculated using the “PCA” and “predict” commands in Stata. These commands create linear composites by standardizing variables to a zero mean and unit variance. Then factor score coefficients are weighted and summed for each factor [53]. Dietary patterns were dichotomized as high adherence (being in the highest quartile of diet scores for each pattern) vs. low adherence (lower three quartiles) [48].

Analysis were performed to assess if there was a modifying effect by gender in the association between SES indicators and the dietary patterns. As there was no evidence of such an effect (p for interaction ≥0.05), analyses were performed for men and women together and adjusted by sex.

As the outcomes of interest (being in the highest quartile of each dietary pattern) were not a rare event (prevalence higher than 10%), the associations between SES and dietary patterns were assessed using Poisson regression models to avoid overestimation of associations [48, 54]. Four different models were built: 1) adjusted for age, sex, year and education; 2) adjusted for age, sex, year and income; 3) adjusted for age, sex, year, education and income and 4) same as Model 3 and including an interaction term for education and income. The latter model was aimed at assessing the modifying effect of income on the association between dietary patterns and education.

## Results

### Sample characteristics

Participants with missing values on the variables used in this study were excluded (S1 Fig). Only 19 participants were excluded from the original sample due to missing information on income or dietary data. Analyses were based on 2476 participants, 56% of whom were women, 72% had low education and 69% had low income (S2 Table).

### Dietary patterns

Three dietary patterns were identified: “snacks and drinks” (composed mainly of soft and energy drinks, salty and sweet snacks, and fruit juice), “fruit and vegetables” (composed mainly of fruit, vegetables and salad), and “fish and rice”. The total variance explained by the three patterns was 29% (Table 1).

**Table 1:** Factor loadings of the principal dietary patterns identified in 2004 and 2013 (n = 2476).

	Dietary pattern		
	Snacks and drinks	Fruit and vegetables	Fish and rice
Soft and energy drinks	0.5217		
Salty snacks	0.5136		
Sweet snacks	0.3788		
Fruit juice	0.2842		
Vegetables		0.6299	
Salad		0.6064	
Fruit		0.3894	
Fish			0.6242
Rice			0.3948
Variance explained (%)	10.1%	9.8%	8.9%

Only foods with factor loadings >0.2 or <-0.2 are shown. Total explained variance by the three components = 28.8%.

### Population characteristics by adherence to patterns

The baseline characteristics by adherence to the three defined dietary patterns are shown in Table 2. Men had a higher adherence to the “snacks and drinks” and “fish and rice” patterns compared to women, but a lower adherence to the “fruit and vegetables” pattern (p<0.001). Individuals with high SES had a higher adherence to the “snacks and drinks” and “fruit and vegetables” patterns, but a lower adherence to the “fish and rice” pattern (p<0.001). Older participants had a lower adherence to the “snacks and drinks” pattern, but a higher adherence to the “fish and rice” pattern.

**Table 2:** Population characteristics by adherence to dietary patterns.

	Snacks and drinks		p-value*	Fruit and vegetables		p-value*	Fish and rice		p-value*
	High (n = 619)	Low (n = 1857)		High (n = 619)	Low (n = 1857)		High (n = 619)	Low (n = 1857)	
Age, mean (SD)	39.2 (10.1)	47.5 (10.6)	<0.001	45.7 (11.0)	45.3 (11.2)	0.514	47.3 (11.1)	44.8 (11.0)	<0.001
Sex (%)									
Men	33.1	66.9	<0.001	20.5	79.5	<0.001	29.2	70.8	<0.001
Women	18.6	81.4		28.5	71.5		21.7	78.3	
Education (%)									
Low	22.4	77.6	<0.001	23.2	76.8	0.001	28.6	71.4	<0.001
High	31.8	68.2		29.8	70.2		15.5	84.5	
Income (%)									
Low	23.4	76.6	0.006	22.4	77.6	<0.001	28.1	71.9	<0.001
High	28.6	71.4		30.8	69.2		18.0	82.0	

* P values for difference in adherence to food pattern by sociodemographic variables were obtained from chi^2^ test for categorical variables and t-test for continuous variables.

Low education: secondary (obligatory), post secondary vocational or lower. High education: polytechnic and university. High income defined as income ≥3,001 Rupees in 2004 and ≥8,001 Rupees in 2013. SD: standard deviation.

### Associations of socioeconomic indicators with dietary patterns

Age and sex-adjusted associations of SES indicators with the dietary patterns are shown in Table 3. As for the “snacks and drinks” pattern, education was not related to adherence to this pattern while individuals with low income tended to have a lower adherence, although associations were not significant [prevalence ratio (PR) 0.93 (0.81–1.07) for low vs. high income]. No evidence was found for an interaction between education and income. Older individuals and women had a lower adherence to this pattern. When adjusting for age, sex, education and/or income, individuals assessed in the 2013 survey had a higher adherence to this pattern compared to those assessed in the 2004 survey.

**Table 3:** Adherence to the different patterns according to socioeconomic indicators assessed by Poisson regression (n = 2476). Model 1: adjusted for age, sex, year and education; Model 2: adjusted for age, sex, year and income; Model 3: adjusted for age, sex, year, education and income; Model 4: same as Model 3 and including an interaction term for education and income. Low education: secondary (obligatory), post secondary vocational or lower. High education: polytechnic and university. High income defined as income ≥3,001 Rupees in 2004 and ≥8,001 Rupees in 2013. Significant associations are indicated in bold.

	Snacks and drinks				Fruit and vegetables				Fish and rice			
	Model 1	Model 2	Model 3	Model 4	Model 1	Model 2	Model 3	Model 4	Model 1	Model 2	Model 3	Model 4
	**0.95**	**0.95**	**0.95**	**0.95**	**1.01**	**1.01**	**1.01**	**1.01**	**1.01**	**1.01**	**1.01**	**1.01**
Age	**(0.94–0.95)**	**(0.94–0.95)**	**(0.94–0.95)**	**(0.94–0.95)**	**(1.00–1.01)**	**(1.00–1.01)**	**(1.00–1.01)**	**(1.00–1.01)**	**(1.00–1.02)**	**(1.01–1.02)**	**(1.01–1.02)**	**(1.01–1.02)**
Sex												
Male	1	1	1	1	1	1	1	1	1	1	1	1
Female	**0.54**	**0.54**	**0.55**	**0.55**	**1.38**	**1.49**	**1.47**	**1.49**	**0.77**	**0.7**	**0.71**	**0.72**
	**(0.47–0.61)**	**(0.48–0.62)**	**(0.48–0.62)**	**(0.48–0.62)**	**(1.20–1.60)**	**(1.29–1.72)**	**(1.27–1.70)**	**(1.28–1.72)**	**(0.68–0.88)**	**(0.62–0.80)**	**(0.63–0.81)**	**(0.63–0.82)**
Education												
High	1		1		1		1		1		1	
Low	0.99		1.02		**0.75**		0.88		**1.56**		**1.29**	
	(0.87–1.14)		(0.88–1.18)		**(0.64–0.87)**		(0.74–1.05)		**(1.29–1.90)**		**(1.05–1.59)**	
Year												
2004	1	1	1	1	1	1	1	1	1	1	1	1
2013	**1.15**	**1.16**	**1.16**	**1.16**	0.96	0.99	0.98	0.98	**0.37**	**0.35**	**0.36**	**0.36**
	**(1.01–1.31)**	**(1.02–1.31)**	**(1.02–1.31)**	**(1.02–1.31)**	(0.84–1.10)	(0.86–1.13)	(0.85–1.12)	(0.85–1.12)	**(0.32–0.43)**	**(0.30–0.41)**	**(0.31–0.42)**	**(0.31–0.42)**
Income												
High		1	1			1	1			1	1	
Low		0.94	0.93			**0.67**	**0.71**			**1.71**	**1.58**	
		(0.82–1.07)	(0.81–1.07)			**(0.58–0.77)**	**(0.60–0.83)**			**(1.45–2.01)**	**(1.32–1.88)**	
Education and income												
High education, high income				1				1				1
High education, low income				0.98 (0.79–1.21)				0.79 (0.62–1.01)				**2.20 (1.57–3.09)**
Low education, high income				1.08 (0.87–1.33)				0.97 (0.77–1.21)				**1.69 (1.23–2.32)**
Low education, low income				0.96 (0.81–1.14)				**0.64 (0.53–0.76)**				**2.34 (1.77–3.09)**
P-total interaction				0.53				0.25				**0.02**

Regarding the “fruit and vegetables” pattern, individuals with low SES had a lower adherence to this pattern in age and sex-adjusted analyses [PR 0.75 (0.64–0.87) for low vs. high education, and 0.67 (0.58–0.77) for low vs. high income]. The association between education and the “fruit and vegetables” pattern was partly attenuated after adjustment for income and became not significant. Income did not modify the association between education and adherence to the “fruit and vegetables” pattern (p for interaction = 0.25). Older individuals and women had a higher adherence to this pattern.

As to the “fish and rice” pattern, individuals with low SES had a higher adherence to the “fish and rice” pattern [PR 1.56 (1.29–1.90) for low vs. high education and 1.71 (1.45–2.01) for low vs. high income]. Associations remained after mutual adjustment for education and income. Income modified the association between the “fish and rice” pattern and education (p for interaction = 0.02), with low income individuals having a higher adherence to the pattern independently of education. Older individuals had a higher adherence to this pattern while women had a lower adherence. When adjusting for age, sex, education and/or income, individuals assessed in the 2013 survey had a lower adherence to this pattern compared to those assessed in the 2004 survey.

## Discussion

This study assessed dietary patterns and their social distribution in an upper middle income country of the African region. Individuals with low SES had a lower adherence to the “fruit and vegetables” pattern but were more likely to consume a traditional diet (“fish and rice” pattern) than their more advantaged counterparts. Moreover, low income individuals had a higher adherence to the “fish and rice” pattern across all education levels. Although associations were not significant, lower income tended to be related to a lower adherence to a western-like pattern characterized by “snacks and drinks”.

### Dietary patterns

The patterns identified in this study were similar to those generally found in other low and middle income settings. For example, the “snacks and drinks” pattern shared several items with the “processed food” and “snacking” patterns identified in Brazil and Africa. These included salty snacks, soda, beer, processed foods [49], fried foods and sweetened products [55]. The “fruit and vegetables” pattern is similar to the “healthy pattern” found in two Iranian studies [56, 57]. Finally, a “balanced” pattern identified in Bangladesh included the same components as the “fruit and vegetables” and “fish and rice” patterns [58]. Our findings support the view that high SES individuals have a lower adherence to “traditional-like” diets, possibly related to the rapid economic development experienced by the Seychelles [38]. We did not assess changes of dietary patterns over time in our population. However, our results suggest that the Seychelles is experiencing the nutrition transition and the population tends to substitute fiber-rich traditional foods with high-fat diets. This is confirmed by data on obesity over time in Seychelles [59].

### Associations of socioeconomic indicators with dietary patterns

The low SES group had a lower adherence to the “fruit and vegetables” pattern, a common finding in both LMICs [11] and HICs [60]. This may be explained by a lack of knowledge regarding the benefits of fruit and vegetable consumption, their high price, and their limited accessiblility and availability [10] due to low local production [61] and large reliance on imported produce.

In this study, low education and low income individuals had a higher adherence to the “fish and rice” pattern, which included two of the traditional staple foods in Seychelles. This result is in line with previous studies conducted in HICs [62, 63] and LMICs [48, 64], where low SES individuals adhere more to a traditional-like diet. This may be partly explained by the fact that high SES individuals tend to choose modern western-like “trendy” foods as a matter of prestige [2], while low SES individuals have lower access to expensive imported foods (other than essential subsidized food items). Thus, they tend to maintain their traditional diet, which generally includes low-cost staple foods, widely consumed and often subsidized to fulfill the minimum caloric needs of a household [65, 66].

In addition, our results suggest that income plays a major role in the substitution of traditional foods with new foods in all educated groups. This result is consistent with previous studies showing that increasing income is related to a substitution of traditional foods with animal-based high fat diets [67].

In parallel with a higher adherence to a traditional pattern, our study suggests that the low SES group had a lower adherence to the “snacks and drinks” pattern. Although non-significant, possibly due to a lack of statistical power, this result may indicate that the Seychelles is at a degenerative diseases stage of the nutrition transition, when foods high in added sugars, fats and soft drinks are more likely to be consumed by the most advtanged sections of society [17, 20, 68]. This is in contrast with our hypothesis that low SES groups would have shown a higher adherence to a western-like diet, due to the rapid economic development of the country. Our results thus confirm that the nutrition transition and the shift of unhealthy diets towards the low SES group occurs at a different pace in different countries. Moreover, our results are in line with the higher prevalence of overweight and obesity among high SES men in the Seychelles [59, 69], although not with the higher prevalence of obesity among low SES women. A possible explanation is that low SES women have a high access to traditional cakes, samoosa, banana or breadfruit chips while high SES individuals have a higher access to “modern” sweet snacks like chocolate bars and chips.

In our study, education and income seem to influence dietary patterns independently, as associations were only modestly attenuated by mutual adjustment. This has been reported previously [70, 71]. Moreover, individuals with a low income tended to stick to a traditional diet irrespective of their educational level, probably because of its lower cost, and it may be considered as a fair option to fulfill the daily caloric requirement although it may lack a number of nutrients (e.g. fiber).

A previous study showed that Seychelles is experiencing a nutrition transition characterized by a decreased consumption of traditional staple foods (fish, rice), beverages (tea), inexpensive home brews, and by an increased intake of meat, poultry and snacks [34]. Our results suggest that this pattern is mostly affecting high SES individuals. However, as experienced in HICs and for other CVRFs in LMICs [72, 73], it is possible that this gradient reverses in the future, thus contributing to increase the burden of NCDs among low SES individuals.

Global efforts for the prevention of NCDs (e.g. the Global Action Plan of the World Health Organization) are proposing prevention strategies aimed at all social strata. However, as our results indicate, the degree of adherence to a “western-like” and “traditional” patterns might not be proportionate in all socioeconomic groups. Moreover, educational interventions alone might not be sufficient, especially in the Seychelles, where income seems to play a more important role to determine dietary consumption.

### Strengths and limitations

This study is based on nationally representative data and represents one of the very few studies assessing socioeconomic differences in dietary intake in an African country. However, some limitations must be considered. First, in order to harmonize education and income across the two surveys, we used dichotomized variables thereby losing information on middle SES groups. Second, comparable dietary information was available for a limited number of foods only and this may limit the identification of precise dietary patterns. Still, the patterns identified were in agreement with those observed in other studies conducted in LMICs, so the loss of information might be small. Third, we combined data from 2004 and 2013 even though the nutrition transition may have progressed during this period. However, the same dietary patterns were found when data were analyzed separately for each year. Fourth, foods that were introduced in the more extensive 2013 questionnaire were not taken into account for this analysis as only food items that were common to the two years were examined. However, our study did not aim to assess changes in diet between years. Fifth, FFQs have been widely used in epidemiological studies since more than 2 decades [43]. However, these instruments are prone to reporting biases, and their validity has been questioned regarding portions measurement and precision [44]. As performed by others [74], we did not assess portion sizes to reduce the burden on participants and limit the lack of precision and number of missing values. We assessed the number of days per week a participant would consume a food item as performed previously [75], although such categorization (with responses ranging from 0 to 7) should not influence the results obtained by PCA to derive dietary patterns. Finally, the small sample size may have decreased statistical power in some of the analysis.

In summary, our study shows that, in a middle income country, low SES individuals have a lower consumption of fruit and vegetables but a higher consumption of traditional foods such as fish and rice, a fair option to fulfil the daily caloric needs. As the Seychelles seems to be at a degenerative diseases stage of the nutrition transition, it is possible that this pattern may reverse along with socioeconomic development and the progress of the nutrition transition. This underlies the need to develop and implement adequate policies to limit unhealthy foods (e.g. taxes) and promote healthy and locally produced foods (e.g. reformulation of foods items regarding fat, sugar or salt, healthy food programs in schools, etc.).

## Supporting Information

S1 Fig Exclusion criteria and final sample included in the study. (DOCX)

S1 Table Food items included in PCA analysis, by frequency of intake. (DOCX)

S2 Table Baseline characteristics of included participants. (DOCX)
