# Supplementary material for: Socioeconomic Differences in Dietary Patterns in an East African Country: Evidence from the Republic of Seychelles
Source: PLoS One. 2016 May 23;11(5):e0155617. doi: 10.1371/journal.pone.0155617 (PMC4877066; doi:10.1371/journal.pone.0155617)
Supplement: S1 Fig — (DOCX) [file pone.0155617.s001.docx]

**Supplementary Figure 1**. Exclusion criteria and final sample included in the study.
