# Supplementary material for: Socioeconomic Differences in Dietary Patterns in an East African Country: Evidence from the Republic of Seychelles
Source: PLoS One. 2016 May 23;11(5):e0155617. doi: 10.1371/journal.pone.0155617 (PMC4877066; doi:10.1371/journal.pone.0155617)
Supplement: S1 Table — (DOCX) [file pone.0155617.s002.docx]

**Supplementary Table 1**. Food items included in PCA analysis, by frequency of intake.

| **Foods included in the PCA** | **2004 Survey (n=1236)** | | **Foods included in the PCA** | **2013 Survey (n=1240)** | |
| --- | --- | --- | --- | --- | --- |
|  | **High intake (%)** | **Low intake (%)** |  | **High intake (%)** | **Low intake (%)** |
| Fruit (e.g. fresh, canned) | 52 | 48 | Fruit (e.g. fresh, canned) | 58 | 42 |
| Vegetables | 76 | 24 | Vegetables | 82 | 18 |
| Starchy roots and tubers (cassava, breadfruit, etc.) | 2 | 98 | Starchy roots and tubers (cassava, breadfruit, etc.) | 4 | 96 |
| Cereals (e.g. cornflakes, hotmeal) | 14 | 86 | Breakfast cereals (e.g. cornflakes) | 18 | 82 |
| Rice | 92 | 8 | Rice | 86 | 14 |
| Salad (e.g. tomato, carrots) | 59 | 42 | Salad (e.g. tomato, carrots) | 54 | 46 |
| Cheese | 14 | 86 | Cheese | 22 | 78 |
| Milk | 25 | 75 | Milk | 22 | 78 |
| Fish | 89 | 11 | Fish (fresh or salted) | 77 | 23 |
| Meat (other than chicken, bacon, sausage, corned beef, ham) | 1 | 99 | Meat (fresh or processed) | 7 | 93 |
| Chicken, bacon, sausage, corned beef, ham | 2 | 98 | Poultry | 6 | 94 |
| Salty snacks (e.g. gato piman, samosa, chips) | 13 | 87 | Salty snacks (e.g. chips, samosa) | 12 | 88 |
| Sweet snacks (e.g. chocolate, ice cream, sweets, cakes) | 10 | 90 | Sweet snacks (e.g. chocolate, ice cream, sweets, cakes) | 15 | 85 |
| Coffee | 12 | 88 | Coffee | 12 | 88 |
| Lemonade, soft drinks | 20 | 80 | Energy drinks or soft drinks | 13 | 87 |
| Juice | 23 | 77 | Juice (fresh) | 33 | 67 |
| Water | 99 | 1 | Water | 99 | 1 |
| Tea | 81 | 19 | Tea | 78 | 22 |

Results are presented as row percentages of consumption. High and low intake represent > or ≤ 4 times per week, respectively. This cut-off was randomly chosen to categorize intake per week.
