# Supplementary material for: Socioeconomic Differences in Dietary Patterns in an East African Country: Evidence from the Republic of Seychelles
Source: PLoS One. 2016 May 23;11(5):e0155617. doi: 10.1371/journal.pone.0155617 (PMC4877066; doi:10.1371/journal.pone.0155617)
Supplement: S2 Table — (DOCX) [file pone.0155617.s003.docx]

**Supplementary Table 2.** Baseline characteristics of included participants.

| **Characteristic** | **(n=2476)** |
| --- | --- |
| Age, mean (SD) | 45.4 (11.1) |
| Sex (%) |  |
| Men | 44.1 |
| Women | 55.9 |
| Education (%) |  |
| Low | 72.3 |
| High | 27.7 |
| Income (%) |  |
| Low | 69.2 |
| High | 30.8 |

High education: polytechnic and university. High income defined as income ≥3,001 Rupees in 2004 and ≥8,001 Rupees in 2013.
